# Differences in Cardiac Effects of Venoms from Tentacles and the Bell of Live *Carukia barnesi*: Using Non-Invasive Pulse Wave Doppler

**DOI:** 10.3390/toxins13010019

**Published:** 2020-12-29

**Authors:** Mark Little, Peter Pereira, Jamie Seymour

**Affiliations:** 1 Emergency Department, Cairns Base Hospital, Cairns, QLD 4870, Australia; Mark.Little@health.qld.gov.au; 2 Australian Institute of Tropical Health & Medicine, James Cook University, Cairns, QLD 4878, Australia; Jamie.seymour@jcu.edu.au

**Keywords:** *Carukia barnesi*, Irukandji, venom, tentacle, Doppler

## Abstract

*Carukia barnesi* was the first in an expanding list of cubozoan jellyfish whose sting was identified as causing Irukandji syndrome. Nematocysts present on both the bell and tentacles are known to produce localised stings, though their individual roles in Irukandji syndrome have remained speculative. This research examines differences through venom profiling and pulse wave Doppler in a murine model. The latter demonstrates marked measurable differences in cardiac parameters. The venom from tentacles (CBV_t_) resulted in cardiac decompensation and death in all mice at a mean of 40 min (95% CL: ± 11 min), whereas the venom from the bell (CBV_b_) did not produce any cardiac dysfunction nor death in mice at 60 min post-exposure. This difference is pronounced, and we propose that bell exposure is unlikely to be causative in severe Irukandji syndrome. To date, all previously published cubozoan venom research utilised parenterally administered venom in their animal models, with many acknowledging their questionable applicability to real-world envenomation. Our model used live cubozoans on anaesthetised mice to simulate normal envenomation mechanics and actual expressed venoms. Consequently, we provide validity to the parenteral methodology used by previous cubozoan venom research.

## 1. Introduction

*Carukia barnesi* was the first of many cubozoan jellyfish known to cause Irukandji syndrome [1,2]. While Huynh et al. [3] showed that it was the principal causative animal in victims demonstrating Irukandji syndrome in Cairns, Australia, other regions of the tropical and subtropical world have identified other jellyfish species. Irukandji syndrome is typified by victims experiencing an innocuous sting while swimming in the ocean followed by delayed truncal pain, hypertension, tachycardia, and agitation among other symptoms and signs of catecholamine excess.

Although most victims settle over hours with supportive treatment, some victims deteriorate and develop severe Irukandji syndrome necessitating intensive care management of life-threatening cardiac failure and intracerebral haemorrhage [3,4,5]. The toxic mechanisms involved remain largely speculative, including the general view that the venom is unlikely to have a direct acting cardiac toxin and instead acts to promote the release of endogenous catecholamines through which the symptoms and signs can be explained. This is indirectly supported by in vivo and in vitro research [6,7,8,9].

**Figure 1:**
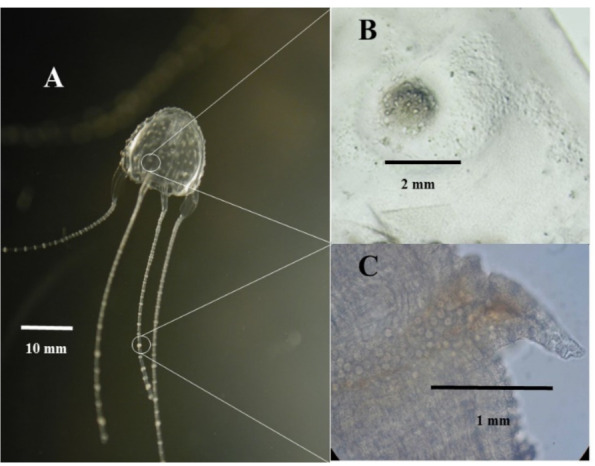
Photomicrographs of (**A**) *Carukia barnesi* with retracted tentacles, (**B**) a wart on the bell, (**C**) a neckerchief on a tentacle.

Morphological descriptions of *C. barnesi* include characteristic clumps of nematocysts on the bell (called mammillations or warts) and on the tentacles (neckerchiefs) (Figure 1).

Although both clumps are populated with the same nematocyst subtypes, they differ significantly in proportions. Morphological descriptions only recognised 2 subtypes of nematocysts, Type I (homotrichous microbasic rhopaloids), and Type II (homotrichous haplonemes) (Table 1, [10]), although a third subtype (microbasic mastigophores) was described in 2010 [4] in *C. barnesi* with larger than 25 mm inter-pedalial distance.

Underwood and Seymour [10] suggested that the differences between bell and tentacle nematocyst concentrations may be ontogenetically related to changing prey targets from invertebrates to vertebrates during the jellyfish’s maturation and growth.

Clinically, Barnes [2] noted that both the bell and tentacles produced local stings when applied to volunteers in his classic paper on causation. Notably, although all his victims developed Irukandji syndrome, both bell and tentacles were applied to each victim, and consequently he did not identify if either or both (bells and tentacles) are responsible for the development of Irukandji syndrome. There are no published papers that clarify this matter, and there have been only nominal attempts to identify differences where case studies provide dermatological descriptions of the sting site. As such, a “string of pearls” appearance, is attributed to the tentacular nematocyst distribution (neckerchiefs), while a single red wheal with localised piloerection (goose pimples) has been inferred to as an encounter with the bell [11]. Furthermore, unsupported clinical opinions attribute significant envenomation to tentacular stings from presumptive exposure to more clumps of nematocysts and hence a greater venom load.

Apart from these convenient dermatographic associations, there are no scientific examinations of the difference between bell and tentacle stings, hence our attempt to examine their cardiac effects using pulse wave Doppler in the murine model described by Seymour et al. [12]. In their study, reconstituted *C. barnesi* venom (CBV, mixed tentacular and bell venoms) was parenterally administered to anaesthetised mice. They observed measurable increases in inotropy and chronotropy preceding cardiac compromise and death, which suggested a model consistent with toxin-induced stress cardiomyopathy.

We postulated that differences in effects between venom from the tentacles (CBV_t_) and bells (CBV_b_) may be quantitatively assessed through non-invasive pulse wave Doppler examination of the cardiac function.

## 2. Results

### 2.1. Venom Profiles

Venom profiling with size exclusion chromatography and SDS-PAGE demonstrates distinct differences between venoms of the bell and tentacles.

#### 2.1.1. Size Exclusion Chromatography (FPLC)

**Figure 2:**
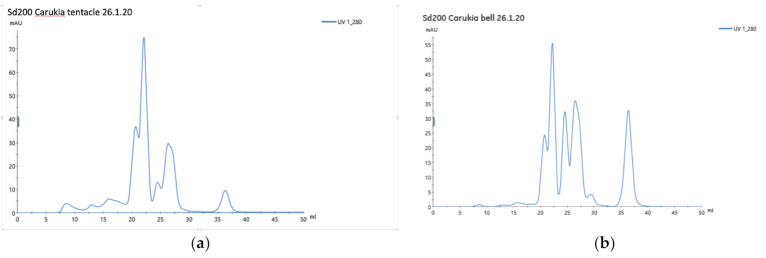
(**a**) FPLC graph for CBV_t_. (**b**) FPLC graph for CBV_b_. Time of elution of the column is displayed on the x axis (min) and UV absorbance on the y axis (%).

Figure 2a,b shows FPLC graphs for CBV_t_ and CBV_b_, and although peaks are similar, they feature obvious differences in height and timing. Of note is the relative reduction in heights at between 5 min and 20 min elution time.

#### 2.1.2. SDS-PAGE

**Figure 3:**
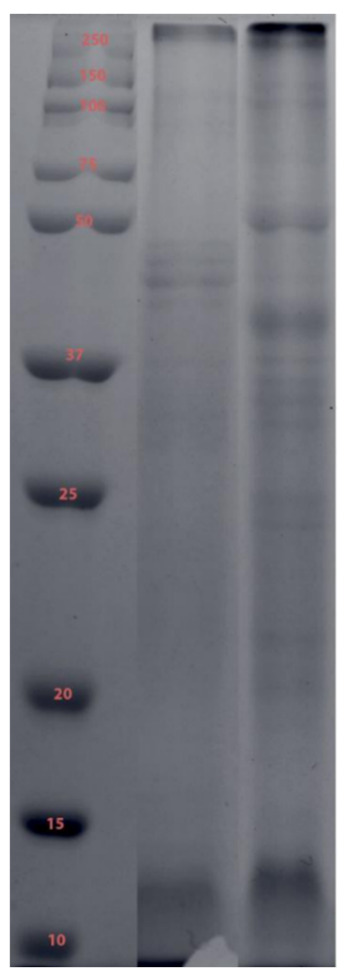
One-dimensional reducing SDS-PAGE analysis of *C. barnesi* venom. Lane 1: molecular mass standards (kilodaltons); lane 2: mature tentacle venom; lane 3: mature bell venom.

There were distinct differences in protein profiles of the venom extracted from the bell and tentacles of the *C. barnesi* (Figure 3). All venom samples contained at least 48 different proteins ranging from 25 to 250 kDa. Venom from nematocysts extracted from mature bells displayed the largest range of sizes and the greatest number of proteins with approximately 60 different recognizable bands. The majority of proteins were less than 100 kDa; however, bell and tentacle venoms shared bands at approximately 100 to 150 kDa. The proteins of highest intensity in both venom sources were found between 75 and 20 kDa.

### 2.2. Pulse Wave Doppler

All mice exposed to tentacular venom (M_tent_) died (mean: 40 min; 95% CL: ± 11 min). All mice exposed to bell venom (M_bell_) remained alive at 60 min, whereupon they were euthanased.

**Figure 4:**
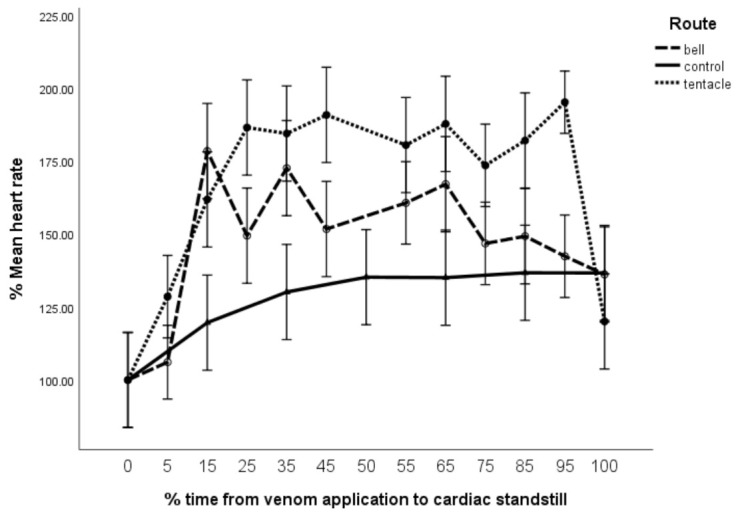
Heart rate (%HR) vs. time (%T). Error bars are 95% confidence limits.

Percentage heart rates for both M_tent_ and M_bell_ increased significantly with time since envenomation (F = 4.9, df = 12 × 73, *p* < 0.005) (Figure 4). Furthermore, M_tent_ demonstrated a higher %HR when compared to M_bell_ and both were significantly higher than the controls (F = 10.05, df = 2 × 73, *p* < 0.005). The latter initially demonstrated tachycardia (peaking at T_15_, ~ 10 min), which trended down to reach equivalence with the control group by T_100_ (60 min). This was significantly different to M_tent_ which maintained its greater tachycardia till T_95_, after which it dropped precipitously below control.

Other than a mild increase in percentage heart rate (%HR) (plateauing from T_50_ to T_100,_ (Figure 4), control mice showed unchanged cardiac parameters for the 60 min duration of their observation, following which they were euthanased. These are provided in the graphs for comparison with parameters from the study mice.

**Figure 5:**
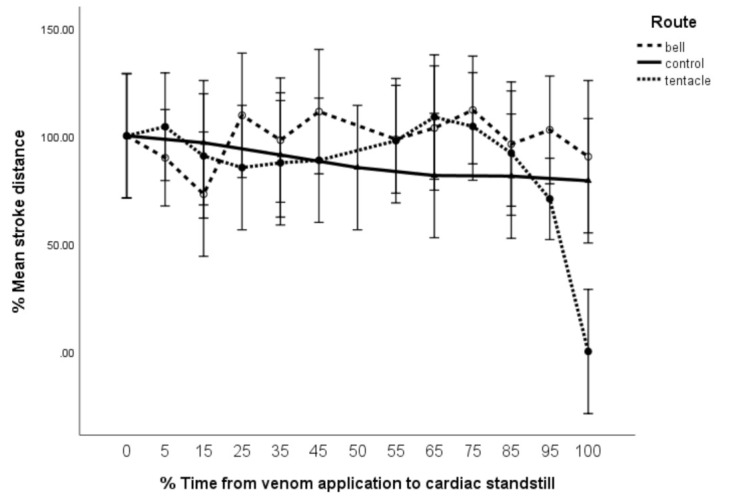
Stroke distance (%SD) vs. time (%T). Error bars are 95% CL.

Both M_bell_ and M_tent_ interacted significantly differently with percentage stroke distance (%SD, F = 1.77, df = 16 × 72, *p* = 0.05) when compared to controls with no significant change noted between M_tent_ and M_bell_ until after T_85_. Following this, M_tent_ deteriorated and reached zero at T_100_ (40 min) (Figure 5). Notably, this reduction in %SD preceded the fall in %HR by 4 min.

**Figure 6:**
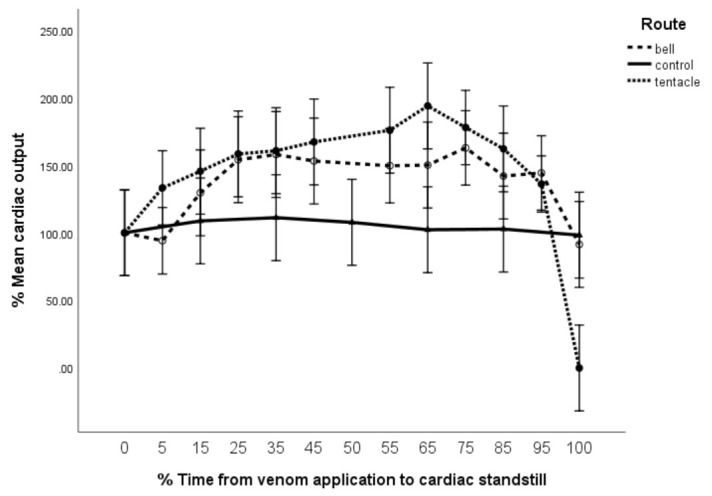
Cardiac output (%CO) vs. time (%T). Error bars are 95% CL. %CO_n_ = 100 × (HR_(n)_ × SD_(n)_ × θ)/(HR_(0)_ × SD_(0)_ × θ), where θ is the cross-sectional area of the mitral valve and (n) and (0) are time_(n)_ and time_(zero)_.

Both M_bell_ and M_tent_ demonstrated significantly elevated percentage cardiac output (%CO) when compared to controls (F = 5.10, df = 2 × 73, *p* = 0.008) (Figure 6). Percentage cardiac output (CO) also increased in both M_tent_ and M_bell_ with time; however, there was a precipitous drop in %CO after T_95_ to 0 in M_tent_ (F = 3.3, df = 16 × 73, *p* < 0.005). In contrast, M_bell_ maintained its increased %CO till %T_95_, after which it appeared to return to equivalence with the control group at 60 min (%T_100_). Being a single data point, this may or may not represent the beginning of deterioration. Note that T_100_ for M_tent_ is 40 min, while T_100_ for M_bell_ is 60 min (note that cardiac output (CO) = stroke volume (SV) × HR, where SV = stroke distance (SD) × valve area [13]).

**Figure 7:**
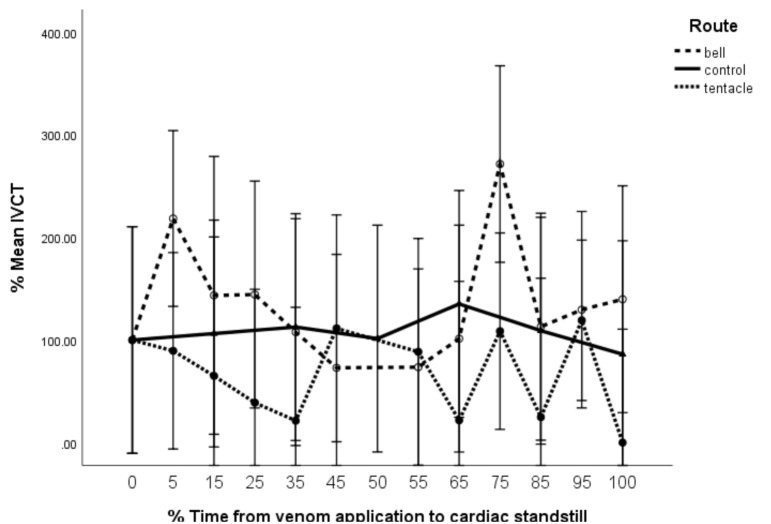
Isovolumetric contraction time (%IVCT) vs. time (%T). Error bars are 95% CL.

When compared to controls, M_bell_ showed no change to %isovolumetric contraction time (%IVCT), while M_tent_ showed statistically significant reduction in %IVCT (F = 5.1, df = 2 × 68, *p* < 0.05 (Figure 7).

**Figure 8:**
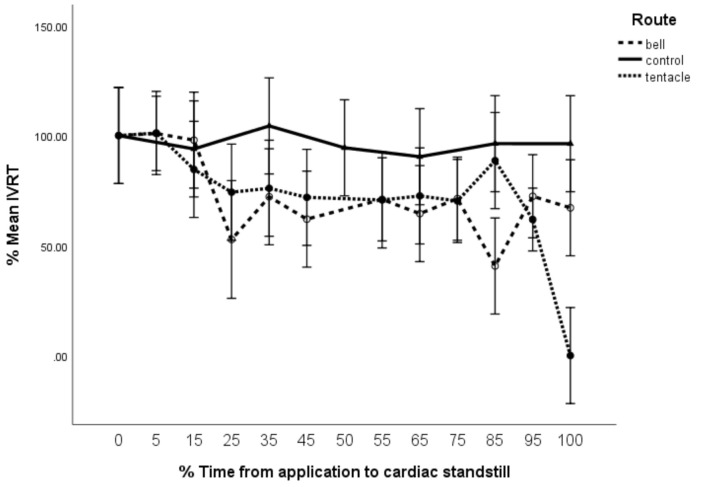
Isovolumetric relaxation time (%IVRT) vs. time (%T). Error bars are 95% CL.

Both M_tent_ and M_bell_ demonstrated significant reductions in isovolumetric Relaxation Time (%IVRT) with time, (F = 4.5, df = 12 × 72, *p* < 0.005), with M_bell_ initially dropping lower than M_tent_ (Figure 8). However, there was significant interaction (F = 2.9, df = 16 × 72, *p* = 0.001) between the time since envenomation and the route of envenomation, with M_tent_ dropping to 0 after %T_95_. For perspective, M_tent_ maintained %IVRT at ~70% for 30 min (%T_15_ to %T_95)_ and then dropped precipitously to zero within 2 min (%T_95_ to %T_100_).

## 3. Discussion

The controls demonstrated mild tachycardia and concurrent reduction in %SD. Although both ketamine and phenobarbitone are known direct myocardial depressants, ketamine offsets this effect by promoting endogenous catecholamine release (a useful characteristic in clinical medicine). We can only speculate if these were direct effects of the anaesthetic or if one was a compensatory response. Nevertheless, the combination maintained the %CO output at 100% throughout the observation period. Importantly, there were no changes in systolic (%IVCT) or diastolic (%IVRT) parameters.

Apart from the mode of envenomation, this study replicated the methodology used by Seymour et al. [12]. As we reproduced cardiac parameter changes, we contend that we have provided evidence of validity to their parenterally administered methodology and, to a lesser extent, other cubozoan venom methodologies.

Many methods have been used in venom preparation for experiments with parenterally administered cubozoan venoms [14]. These have been summarised by Yanagihara and Shohet and show marked variation in protein compositions, casting some doubt on the efficacy of the test venoms and outcomes [14]. As mentioned in the previous paragraph, we used the same methodology as described by Bloom [15] and Carrette [16], and consequently this research has additionally provided validation for the venom preparation method described.

This study demonstrates statistically significant differences in measured cardiac effects between venoms from nematocysts on the bell (CBV_b_) compared to venoms from nematocysts on the tentacles (CBV_t_) of *C. barnesi*. Of importance, CBV_b_ did not cause cardiac compromise or death in the M_bell_ group in mice at 60 min post-exposure, whereas CBV_t_ resulted in death in all mice in the M_tent_ group with exposure to a single neckerchief (mean: 40 min; 95% CL: ± 11 min). Although our portrayal of endpoints appears equivalent between the M_tent_ and M_bell_ groups, the actual time difference is a substantial 20 min; for further emphasis, the M_bell_ group ran for 50% longer with no evidence of cardiovascular collapse and trended to normalisation of cardiovascular function. It is feasible that had the experiment run for an even longer period, the M_bell_ group may have registered some fatalities, but 60 min was an ethical and experimental time limit.

The M_bell_ group showed a rapid increase in %HR (Figure 4) peaking at 175% at T_15_ (~10 min) trending down to the control group at T_100_. This group demonstrated an associated reduction in the %IVRT to 80%, which indicates an improved efficiency in ventricular relaxation. Significantly, there was no accompanying reduction in %IVCT, nominally suggesting no increase in ventricular contraction. As we did not assess aortic valvular flow, we were unable to provide other measures of ventricular contractility such as peak aortic velocity and aortic rise time as described by Seymour [12], which would provide a more complete assessment of systolic function and inotropy. Despite their absence, our findings do not demonstrate any increase in inotropy, suggesting that the improved ventricular relaxation may just be an accompanying necessity of the observed moderate increase in %HR.

In contrast to the M_bell_ group, all M_tent_ mice died before the 60 min endpoint. Clearly, the venoms differ significantly, with a lethal effect conferred by tentacular venom. Both the FPLC and SDS gel demonstrate different peaks and bands, respectively, between the two sources. Although there are other differences evident, our FPLC graphs (Figure 2a,b) show greater prominence of the 7–20 min elution peaks in CBV_t_ when compared to the CBV_b_. These peaks have gained attention in *C. fleckeri* fractionated venom research, suspected to contain the lethal components responsible for box jellyfish fatalities [17,18]. We can only speculate that these peaks may be pertinent to our observed difference in lethality. Further research with fractionated CBV_t_ is intended to examine lethal fractions.

For most of the observed period, the %SD (effectively, the stroke volume) remained the same for both M_tent_ and M_bell_ groups and consequently the increase in %CO (Figure 6) is attributable purely to the observed increased chronotropy.

For consistency across the figures, Figure 4 shows M_tent_’s T_100_ based on the loss of %CO rather than the ECG waveform loss (as justified in Section 5.3 of the Materials and Methods section). Had Figure 4 been presented as %HR over 60 min observation instead, not only would M_tent_ have showed the same larger increase in %HR, but it would also have showed a stark reduction in %HR at T_60_ (40 min) to below 50% followed by asystole till T_100_, while both M_bell_ and control graphs would have remained unchanged.

Furthermore, it is significant that the loss in pulse wave Doppler (PWD) waveform (and hence %CO) preceded the reduction in heart rate by 4 min (Figure 4, Figure 5 and Figure 6). Clearly, this represents a failure of contraction rather than the loss of electrical activity.

Additional support for this may be found in comparing parameter progression between Figure 4, Figure 5 and Figure 6. It is notable that when M_tent_ demonstrates a reduction in %SD after T_75_ (Figure 5), there is an accompanying increase in %HR (Figure 4), peaking at T_95_. During this period, %CO is maintained (Figure 6). This may be explained by a reduction in myocardial contractility (fall in %SD), which is compensated for by an increase in %HR between T_75_ and T_95_ (over 8 min). This compensation is overwhelmed at T_95_, following which there is a precipitous fall in %CO at T_100_.

While we nominally noted a widening of the QRS complex accompanying the PWD changes, the authors intend to formally assess ECG parameters (including QRS morphology) in an attempt to examine ion channelopathies that may implicate causation. Our QRS observations may be consistent with Yanagihara and Shohet’s findings of QRS prolongation in that they relate to K^+^ leakage across cell membranes from venom-induced pores in their in vitro model [14]. Ideally, this could have been tested in our experiment, had we measured serum K^+^ changes. However, the need for repeat samples would have required a larger animal model that has a significantly larger blood volume than the mouse’s 2 mL to ensure that repeated sampling did not confound our PWD cardiac assessment.

Unlike the M_bell_ group, the M_tent_ group shows significant reduction in %IVCT as well as the same improved efficiency in ventricular relaxation (reduced %IVRT) (Figure 7 and Figure 8). This suggests that ventricular contraction was more time-efficient for the same ventricular stroke volume, equating to measurable evidence of increased inotropy in this group. Seymour et al. [12] describe similar changes—increases in inotropy and chronotropy with reconstituted CBV, which were promoted as consistent with toxin-induced stress cardiomyopathy from sustained catecholaminergic activity. With this research, we ratify their findings and further refine the position that the measured cardiac decompensation from the *C. barnesi* venom appears to be due to CBV_t_ alone.

This experiment achieved its aim to solely examine the difference between tentacular and bell envenomation. The following paragraphs speculate as to why these differences exist and how they are achieved. These can be separated into differences in the volume of venom delivered, differences in the potency of delivered venom, and differences in the effectiveness of venom delivery.

It appears that all immature cubozoans have warts, but only *Carybdea* species retain these (albeit dwindling numbers) into maturity. Underwood and Seymour [10] measured between 60 and 220 warts on *C. barnesi* bells, the higher numbers associated with juvenile animals which feed solely on invertebrate crustaceans. Notably, these animals had stubby, developing tentacles and an absence of neckerchiefs. Our personal observation is that these immature animals manoeuvre into the path of their prey to effect their sting. As they mature and develop their (clear) tentacles and (visibly prominent) neckerchiefs (Figure 1a), they appear to use these to catch larval fish by twitching their extended tentacles (note that Figure 1a depicts a *C. barnesi* with contracted tentacles, which, when extended, separate the individual neckerchiefs by up to 10 cm). It is promoted that the twitching of the separated neckerchiefs mimics the movement of their prey and this “angling” appears to entice larval fish into fatally attempting to feed on the neckerchiefs.

The inference from their findings is that the change in diet with maturity is achieved by ontogenetic changes to venom, which they ascribe to differences in populations of nematocyst subtypes (Type I and Type II) between warts and neckerchiefs (Table 1). It is pertinent that our M_bell_ model involved the application of half the nematocysts of the bell’s surface and consequently half of the bell’s total nematocysts. This would also constitute a maximum dose in human envenomation. Despite this large exposure, there were no M_bell_ fatalities and only moderate elevations in HR. In contrast, the M_tent_ group was uniformly lethal after exposure to just a single neckerchief. It may be that a neckerchief contains vastly greater numbers of nematocysts than 30+ warts and can hence deliver a greater venom load. As yet, there are no studies into nematocyst density per clump.

Alternatively, the difference in lethality may be explained by a difference in venom composition within the same nematocysts that populate both groups (in different proportions). Further research is required to examine this.

Finally, it is possible that the difference in lethality between M_tent_ and M_bell_ groups could be explained purely by mechanical problems with venom delivery. For instance, it may be that tentacular nematocysts successfully penetrated and delivered venom into the dermis while bell nematocysts failed to do so. Cutaneous histological sections would have assisted in answering this possibility; however, this was not considered in the experiment’s design. In support of effective venom delivery from bell nematocysts, we can refer to the Barnes’ original paper where he described an observed cutaneous reaction to bell exposure which was separate from the tentacular reaction [2].

Our M_bell_ methodology simulates a worst-case scenario with an adult human vs. bell encounter. That it does not result in fatality nor reduce the measured %CO in a mouse when the comparative dose/kg is at least 3000× greater than that with a human victim (24 g mouse vs. 70 kg human) is significant. Other than the remote possibility that this may be explained by mice having an innate resistance to CBV_b_ when compared to humans, this would strongly suggest that the bell is not causative in the cardiac decompensation encountered in severe Irukandji syndrome.

Further extrapolation from a 20 g mouse to a 70 kg human suggests that the bell of *C. barnesi* may not cause any significant clinical catecholaminergic effects and may not be expected to produce any significant clinical systemic envenomation. This, of course, can only be ascertained through verifiable human exposure studies.

The bell’s apparent lack of significant effect may explain the wide variation in clinical severity described by Huynh et al. [3], where some proven *C. barnesi* stings were associated with no systemic symptoms of envenomation. It would also be consistent with other researchers’ ascription of bell exposure to mild stings [11]. It also follows from this that encounters with juvenile *C. barnesi* with poorly developed tentacles are more likely to induce minor stings.

### Limitations

This study was an in vivo murine study, with only 9 mice examined and only 3 *C. barnesi* animals used. We have no way of knowing if there was equivalence in venom dosage between tentacle and bell groups when compared to the parenteral administration of reconstituted venoms, where protein concentrations are measured and dosage can be accounted for. We were careful to expose only a single neckerchief to each mouse in the tentacle group, which is likely to be a comparatively small exposure in a marine environment. In contrast, the application of up to half the bell would constitute a maximum exposure in a similar marine environment.

We were limited to one PWD probe per mouse and we elected to apply this to examine the mitral valve where we could obtain both diastolic and nominal systolic parameters of ventricular function. As such, we did not examine the aortic waveform, where a more complete measure of strength of ventricular contraction and systolic function can be obtained through additional parameters.

## 4. Conclusions

We demonstrate that in the murine model, there are significant differences in toxicity between venoms from the bell and tentacles of *C. barnesi*. In particular, we demonstrate that only venom from the tentacles produces lethal cardiovascular demise in mice. It is noteworthy that despite comparatively large envenomation from the bell, there was no cardiac compromise and no associated deaths. This strongly suggests that CBV_b_ is not implicated in severe Irukandji syndrome.

Furthermore, this large exposure produced only a temporary moderate elevation in %HR (and %CO), which in a dose/kg extrapolation would be unlikely to cause significant systemic effects in human envenomation. Consequently, although bell exposure is known to cause a local sting [2], we provide some evidence that Irukandji syndrome from *C. barnesi* may occur solely from tentacle exposure.

Finally, this research is the first to demonstrate that stings from live cubozoan animals can reproduce similar cardiovascular changes previously described with the parental administration of their venoms. As such, this research provides verification of the parenteral methodology of in vivo cubozoan venom research.

## 5. Materials and Methods

Three live specimens of *C. barnesi* were collected from the waters around Double Island (25°55′00′′ S, 153°11′00′′ E), Northern Australia, using light traps. Their inter-pedalial sizes were 15 mm, 15 mm, and 11 mm. The specimens were housed in seawater containers and applied to the study mice within 24 h of capture.

### 5.1. Venom Profiling

Tentacles were manually excised from the bells at the base of the pedalia. Both tentacles and bells underwent nematocyst isolation and venom extraction and lyophilisation separately using established techniques [15,16]. Reconstituted venoms (CBV_b_ and CBV_t_) were then profiled using size exclusion chromatography (FPLC) and SDS-PAGE.

#### 5.1.1. Size Exclusion Chromatography (FPLC)

Between 200 μL and 500 μL of reconstituted CBV_b_ and CBV_t_ were passed through a 0.22 μm filter and individually analysed using an AKTA^TM^ fast performance protein liquid chromatography (GE-Healthcare Australia and New Zealand, Sydney, Australia), at a flow rate of 0.3 mL/min and wavelength set at 280 nm (Superdex 200 TM 10/300GL; Tricorn; 13 μm, 10 mm × 200 mm). Degassed Dulbecco’s phosphate-buffered saline was used as a running buffer to fractionate the venoms for venom profile generation.

#### 5.1.2. SDS-PAGE

Isolated CBV_b_ and CBV_t_ samples were run on a 4–20% precast polyacrylamide gel at 150 V. Bio-Rad Kaleidoscope Precision Protein Standards (Bio-Rad Laboratories Pty. Ltd, Sydney, Australia) were used for comparison of molecular weights. Gels were then stained with 16% Fast Blue/20% acetic acid solution for 1 h. Excess stain was removed by submerging the gel overnight in 10% acetic acid. Banding patterns were visually assessed for similarities and differences between sample types.

### 5.2. Study and Control Mice

Ten-week-old female laboratory mice (strain BALB/c), bred by the Animal Resources Centre (www.arc.wa.gov.au) and maintained at the James Cook University (JCU) Mouse House were utilised for this research (Their sizes are provided in Table 2).

#### 5.2.1. Controls

Three mice were anaesthetised with intraperitoneal injections of 4 mg ketamine (mean: 170 mg/kg, range: 129–203 mg/kg) and 0.2 mg phenobarbitone (mean: 8.5 mg/kg, range: 6.5–10.2 mg/kg), after which they were weighed (mean: 24.4 g, range: 19.7–30.9 g) and then monitored. All animals remained fully anaesthetised for 60 min without additional anaesthetics, following which they were euthanased with a fatal dose of pentobarbital (3.2 mg) via intracardiac injection. These controls established a baseline for anaesthetised mice and demonstrated maintenance of all measured cardiac parameters for the duration of 60 min and are included in the results graphs. In all the graphs, the cardiac parameters are controlled against themselves at T_0_.

#### 5.2.2. Study Mice

On the day of the experiment, 6 mice were anaesthetised sequentially. When sufficiently anaesthetised as determined by absence of response to firm tail pressure, the mice were weighed (mean: 23.8 g, range: 22.7–25.9 g) and then placed prone on a warmed (37 °C) monitoring mat (Indus Instruments Rodent Surgical Monitor, TX, USA), where ECG and heart rate (HR) were continuously monitored. Using the technique described by Seymour et al. [12], a 10 MHz Doppler probe (Indus Instruments Doppler Flow Velocity System, TX, USA) was focused on the mitral valve and Doppler sonograms of blood flow were recorded for analysis. Each sonogram describes the sequentially collected velocities of individual blood cells passing through the aortic valve. The waveform produced can be analysed to reliably measure indices of cardiac function [13,19].

Depilation of the abdomen was achieved with application of the Nair Sensitive Hair Removal Cream for 5 min. Two pieces of clear office adhesive tape (equivalent to Cellotape) were placed on the depilated abdomen to effect a 1 cm gap between them.

Following this, either (a) a live *C. barnesi* tentacle was stretched across the 1 cm gap (visibly amounting to exposure to a single neckerchief), or (b) a live *C. barnesi* bell was gently pressed into the gap. Both were applied for 1 min before removal. Data were obtained until there was an absence of measurable Doppler complexes (assumed loss of %CO) or the 60 min duration was completed, whereupon the surviving mice were euthanased with a fatal dose of pentobarbital (3.2 mg via intracardiac injection). Like the controls, none of the study mice required further anaesthesia for the 60 min duration of the study.

### 5.3. Waveform Analysis

Analysis of the waveform occurred from %T_0_, defined as time from application of nematocysts to the abdomen. During the period of observation, some mice demonstrated persistence of electrical activity despite a loss of measurable %CO. As the purpose of this research was to examine cardiac dysfunction, it was decided that the endpoint %T_100_ was defined by 60 min observation or the loss of measurable %CO.

On identifying %T_100_, the best four contiguous waveforms at 10% intervals were selected (%T_05_–%T_95_) and the data points on each waveform were manually identified and mapped into the Indus Instruments Signal Analysis software (Doppler Signal Processing Workstation V1.625, Indus Instruments, Webster, TX, USA). This provided the cardiac indices tabled below (Table 3) and graphed in the results.

### 5.4. Statistical Analysis

The effect of time since nematocyst application on various dependent cardiac parameters (such as heart rate, stoke distance, etc.) were determined using a repeated measures one-way analysis of variance. In cases where sphericity was violated, Greenhouse–Geisser correction was applied. All analysis was performed using IBM SPSS Statistics V25. Results of all analyses of variance are displayed as the F statistic, “F = ***”, degrees of freedom for the independent and error term as, e.g., “df = 2 × 12”, and the probability of the effect as “*p*” (in summary, F = “”, df = “”, *p* = “”).

## Figures and Tables

**Table 1:** Nematocyst populations on tentacles and bells [10].

	Homotrichous Microbasic Rhopaloids (Type I)	Homotrichous Haplonemes (Type II)
Bell	31%	69%
Tentacles	98%	2%

**Table 2:** Controls and study mice.

	No	Weight
Controls	3	Mean: 24.4 g, range: 19.7–30.9 g
Bell	3	Mean: 24.5 g, range: 22.7–25.9 g
Tentacle	3	Mean: 23.0 g, range: 22.7–23.6 g

**Table 3:** Cardiac indices from pulse wave Doppler. Further details on these can be found in Seymour et al. [12].

SD	Stroke Distance	Measured Area Under the Pulse Wave Doppler Sonogram (Directly Proportional to the Volume of Contraction (Stroke Volume)).
CO	Cardiac output	Extrapolated from the SD and directly proportional to the ventricular stroke volume and the heart rate.
IVCT	Isometric ventricular contraction time	IVCT: time from initiation of ventricular contraction to aortic valve opening. In the presence of reduced SD, an increased IVCT reflects dysfunctional ventricular contractility (systolic dysfunction).
IVRT	Isometric ventricular relaxation time	IVRT: Time from end of ventricular contraction and mitral valve opening. Increased IVRT reflects dysfunctional ventricular relaxation (diastolic dysfunction).
